# MicroRNA-140-5p regulates osteosarcoma chemoresistance by targeting HMGN5 and autophagy

**DOI:** 10.1038/s41598-017-00405-3

**Published:** 2017-03-24

**Authors:** Yichen Meng, Rui Gao, Jun Ma, Jianquan Zhao, Enjie Xu, Ce Wang, Xuhui Zhou

**Affiliations:** Department of Orthopedic Surgery, Changzheng Hospital, Second Military Medical University, 415 Fengyang Road, Shanghai, 200003 P. R. China

## Abstract

Chemotherapy is an important treatment modality for osteosarcoma. However, it often fails because of chemoresistance, especially multidrug resistance. Previously, we found several genes were involved in chemoresistance development. In this report, we used high-throughput microRNA (miRNA) expression analysis to reveal that expression of miR-140-5p was associated with chemosensitivity in osteosarcoma. The exact roles of miR-140-5p in the chemoresistance of osteosarcoma were then investigated, we found that knockdown of miR-140-5p enhanced osteosarcoma cells resistance to multiple chemotherapeutics while overexpression of miR-140-5p sensitized tumors to chemotherapy *in vitro*. Moreover, *in vivo*, knockdown of miR-140-5p also increased the osteosarcoma cells resistance to chemotherapy. Luciferase assay and Western blot analysis showed that HMGN5 was the direct target of miR-140-5p which could positively regulated autophagy. Silencing these target genes by siRNA or inhibition of autophagy sensitized osteosarcoma cells to chemotherapy. These findings suggest that a miR-140-5p/HMGN5/autophagy regulatory loop plays a critical role in chemoresistance in osteosarcoma. In conclusion, our data elucidated that miR-140-5p promoted autophagy mediated by HMGN5 and sensitized osteosarcoma cells to chemotherapy. These results suggest a potential application of miR-140-5p in overall survival, chemoresistance prognosis and treatment.

## Introduction

Osteosarcoma is the most common malignant bone tumor^1^. The survival outcomes remain unsatisfactory since recurrences are common due to the development of invasion, distant metastasis and chemoresistance. Chemoresistance, both intrinsic and acquired, is a main cause of recurrences or failure of current treatment^2^. Up to date, several factors contributed to the development of chemoresistance including genetic alterations^3^, altered drug accumulation^4^, drug-target amplification^5^, and autophagy^6^. Many cellular signaling pathways have been studied to explore the mechanism of chemoresistance of osteosarcoma. The autophagy signaling pathway has been shown to be one of the most important regulatory networks^7^.

Autophagy, a cytoprotective process, facilitates cell survival by sustaining energy production under cellular stresses conditions like metabolic stress, hypoxia, and chemotherapy. A number of studies have shown a critical role for autophagy in cancer development, especially chemoresistance^8–10^. The anticancer agents could induce autophagy in various tumor cells^11, 12^ and preclinical studies have shown that autophagy inhibition restored chemosensitivity^13^. Totally, autophagy was a drug resistance mechanism in cancer. However, the upstream regulatory mechanism of autophagy is still unclear. High mobility group nucleosome binding domain 5 (HMGN5), which is a kind of ubiquitous nuclear proteins, expressed widely in eukaryotes^14^. An increasing level of HMGN5 mRNA and protein was observed in prostate cancer^15^, adenocarcinoma^16^, squamous cell carcinoma^17^, and breast cancer^18^. More importantly, our previous study demonstrated that HMGN5-mediated autophagy contributed to chemoresistance in osteosarcoma cell lines U2-OS and MG63^19^. However, the mechanism of activating HMGN5-mediated autophagy still remains largely unknown. MicroRNAs (miRNAs) are a class of small (about 22 nucleotides) noncoding RNAs that regulate gene expression by binding to the 3′ untranslated region (3′ UTR) of their target mRNAs, modulating mRNA stability and protein expression at the post-transcriptional level. Aberrant expression of miRNAs has been implicated in the mechanism of chemoresistance^20–22^ and several miRNAs have been identified in osteosarcoma tissues and cell lines^23, 24^.

Recently, miR-140-5p has been found to regulate ovarian cancer growth^25^, stem cell differentiation^26^ and cell autophagy^27^. Moreover, miR-140-5p has been shown to inhibit transforming growth factor β receptor 1 in hepatocellular carcinoma, and overexpression of miR-140-5p could suppress hepatocellular carcinoma growth and metastasis^28^. However, a higher miR-140-5p expression was observed in breast cancer tissue compared with adjacent normal tissue^29^, suggesting that it could promote tumor formation in breast cancer.

Taken together, previous studies demonstrated that miR-140-5p could be both tumor suppressor and promoter and the biological response of cancer cells to miR-140-5p may depend on particular cell type and other factors that has not been defined yet. miR-140-5p was also detected in bone cells and could inhibit BMP2-mediated osteogenesis in human mesenchymal stem cells^30^. However, the exact role of miR-140-5p in osteosarcoma and chemoresistance remains elusive.

In this study, we investigated the expression of miR-140-5p in osteosarcoma tissues and cell lines. We found that osteosarcoma samples frequently featured lowly expressed miR-140-5p comparing to the normal tissue counterparts. A declined expression of miR-140-5p was also observed in human osteosarcoma tissues in the chemoresistent group. Further study showed that knockdown of miR-140-5p promoted chemoresistance of osteosarcoma cells, while overexpression of miR-140-5p promoted chemosensitive. In addition, HMGN5 was identified as the target gene of miR-140-5p in osteosarcoma and HMGN5-mediated autophagy played an important role in the miR-140-5p-mediated chemoresistance. All these results indicate that miR-140-5p and its downstream target gene HMGN5 can be used to prevent and treat the osteosarcoma chemoresistance in the future.

## Results

### miR-140-5p lowly expressed in osteosarcoma tissues after chemotherapy

**Figure 1:**
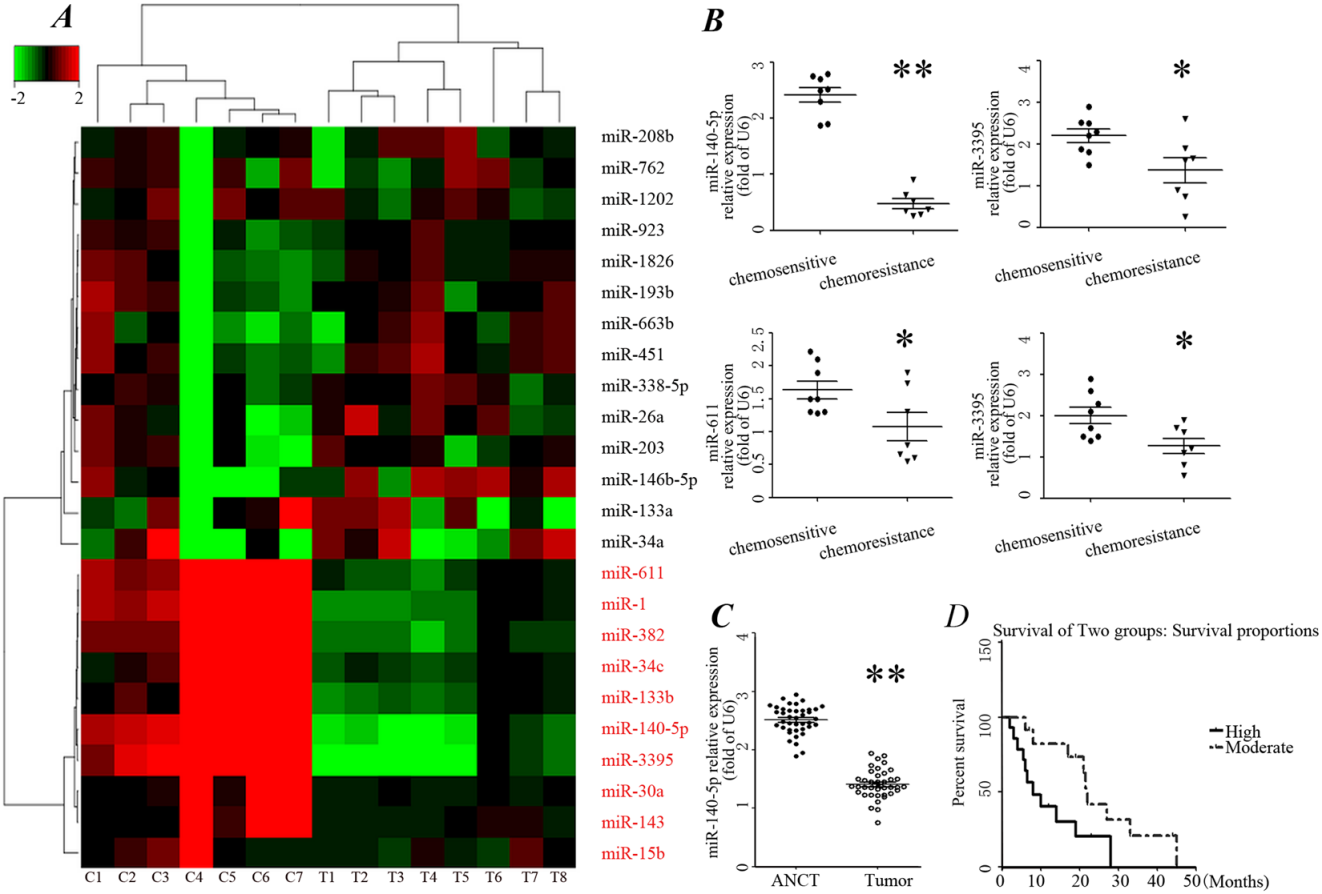
miR-140-5p lowly expressed in chemoresistance chort. (**A**) Heatmap representation of differentially expressed microRNAs in the chemoresistant osteosarcoma cases and chemosensitive osteosarcoma cases. Rows, miRNA; columns, independent biological replicates. Downregulated microRNAs are shown in red, while upregulated microRNAs are shown in green. (**B**) Differentially expressed microRNAs between chemoresistant osteosarcoma cases and chemosensitive osteosarcoma. Expression of miR-140-5p (**C**) in 40 paired osteosarcoma and adjacent normal bone tissues (ANCT). (**D**) Kaplan-Meier analysis for overall survival in 40 osteosarcoma patients in low- and moderate- groups based on miR-140-5p expression levels.

To screen miRNAs that are potentially involved in chemoresistance, miRNA microarray analysis was carried out with a chemoresistance cohort (n = 8, T) and tumor stage-matched chemosensitive control cases (n = 7, C). Totally, 24 miRNAs showed significant differential expression, among which 9 miRNAs were down-regulated and 15 miRNAs were up-regulated (Fig. 1A). miR-140-5p, miR-3395, miR-611 and miR-1 was the top four down-regulated miRNAs in these down-regulated miRNAs. To further confirm the miRNA microarray results, qRT-PCRs were performed and showed that miR-140-5p frequently lowly expressed in chemoresistance cohort (Fig. 1B). However, the other three downregulated miRNAs did not consistently lowly expressed in chemoresistance group. These results suggested that miR-140-5p played an important role in chemoresistance and further functional study was conducted to prove the correlation between miR-140-5p and clinical outcomes.

### miR-140-5p is down-regulated in human osteosarcoma tissues and correlates with multiple clinical features

To further determine whether miR-140-5p expression correlates with clinical outcomes in patients, we tested the expression of miR-140-5p by qRT-PCR in 40 pairs of tumor tissues in Table 1. We analyzed the associations between clinicopathologic characteristics and miR-140-5p expression levels in 40 patients. Totally, the relative level of miR-140-5p expression in osteosarcoma tissues (mean ± SD: 1.36 ± 0.02) was significantly lower than that in matched adjacent nontumor tissues (ANCTs) (mean ± SD: 2.52 ± 0.03; p < 0.001, Fig. 1C). Furthermore, the median value of miR-140-5p expression was used as the cut-off point for dividing samples into two groups: moderate expression group (>median, n = 20) and low expression group (<median, n = 20). We found a statistically significance between miR-140-5p expression and some clinical features of osteosarcoma (Table 1), including tumor size (p = 0.029), clinical stage (p = 0.016), recurrence (p = 0.023) and distant metastasis (p = 0.016). No significant difference was observed between the expression of miR-140-5p with patients’ age (p = 0.945) and gender (p = 0.058).

**Table 1:** Relationship between expression of miR-140-5p and clinicopathologic characteristics of osteosarcoma.

Variables	Cases (n)	miR-140-5p		χ2	P
		moderate	Low		
Total	40	20	20		
Age (y)					
<18	28	12	16	0.005	0.945
≥18	12	5	7		
Gender					
Male	32	17	15	0.625	0.058
Female	12	3	5		
Recurrence					
No	19	9	10	5.199	0.023
Yes	21	3	18		
Stage					
I	9	5	4	5.783	0.016
II-III	31	5	26		
Size of tumor (mm)					
<50	15	8	7	4.748	0.029
>= 50	25	5	20		
Distant metastases					
Yes	30	6	24	5.714	0.016
No	10	6	4		

Kaplan-Meier analysis revealed that patients with moderate miR-140-5p expression survived significantly longer than those with low miR-140-5p expression by using the log rank test (Fig. 1D, p = 0.027). Our results suggested that miR-140-5p is closely correlates with multiple clinical features in osteosarcoma.

### miR-140-5p regulated the sensitivity of osteosarcoma cells to chemotherapeutic agents *in vitro* by targeting HMGN5

**Figure 2:**
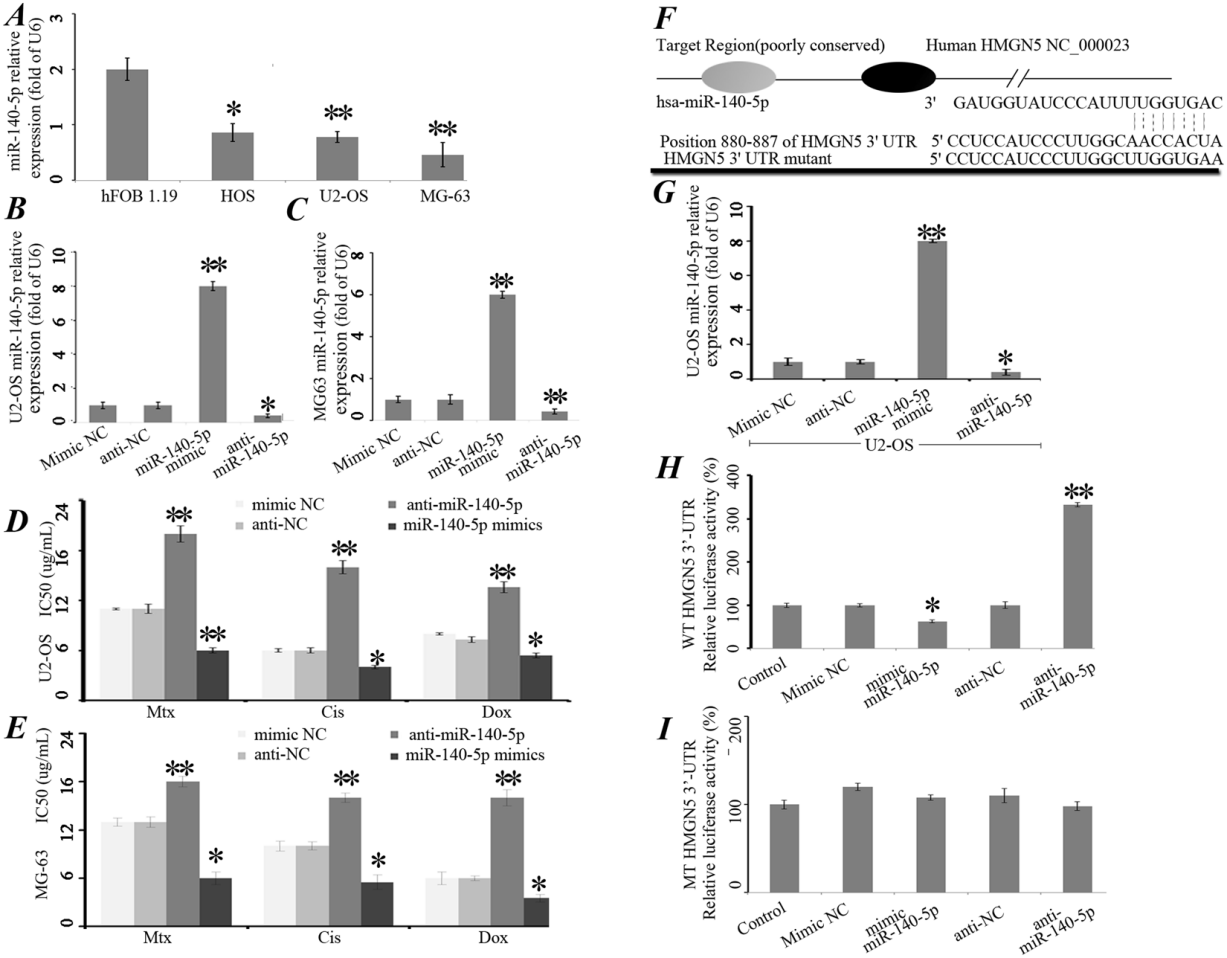
miR-140-5p regulated the sensitivity of osteosarcoma cells to chemotherapeutic agents *in vitro* by targeting HMGN5. (**A**) Endogenous expression levels of miR-140-5p in the human osteoblastic cell line, hFOB 1.19, and three osteosarcoma cell lines were determined by qRT-PCR and normalized to those of U6 snRNA. Each bar represents the mean of 3 independent experiments. *P < 0.05, **P < 0.01. Expression of miR-140-5p was detected by qRT-PCR after transfected with mimic NC, miR-140-5p mimics (50 NM), anti-NC or anti-miR-140-5p (100 NM) in U2-OS (**B**) and MG-63 (**C**) cells. miR-140-5p up-regulated the chemoresistence of osteosarcoma cells to chemotherapeutic agents in U2-OS (**D**) and MG-63 (**E**) cells *in vitro*. (**F**) The target genes of miR-140-5p were predicted using bioinformatic analysis. (**G**) Expression of miR-140-5p was detected by qRT-PCR after transfection of U2-OS cells with mimic NC, miR-140-5p mimics, anti-NC or anti-miR-140-5p. (**H**) Dual luciferase assays were performed in U2-OS cells after cotransfection with HMGN5 3′-UTR plasmids and anti-miR-140-5p or miR-140-5p mimics. (**I**) Luciferase construct containing mutant target site of the HMGN5 3′-UTR was generated and transfected as indicated. *P < 0.05, **P < 0.01.

To confirm the results in Fig. 1, miR-140-5p expression was examined in four osteosarcoma cell lines using quantitative real-time PCR (qRT-PCR). qRT-PCR results showed that compared with hFOB 1.19 cells, miR-140-5p was significantly down-regulated in osteosarcoma cell lines (Fig. 2A).

In addition, U2-OS and MG-63 cells were transiently transfected with mimic NC, miR-140-5p mimics, anti-NC or anti-miR-140-5p (Fig. 2B,C), and the expression of miR-140-5p was studied after transfection. Cell proliferation assays were performed to determine cell growth curve and 50% inhibition of growth (IC50) values (Fig. 2D,E). Knockdown of miR-140-5p dramatically enhanced the IC50 values for these three chemotherapeutic agents in U2-OS and MG-63 cells (Fig. 2D,E).

To predict the target genes of miR-140-5p, bioinformatics analysis were performed and the results showed that the 3′-UTR region of HMGN5 were identified as the binding sites for miR-140-5p (Fig. 2F). Mimic NC, miR-140-5p mimics, anti-NC or anti-miR-140-5p were transfected into the U2-OS cell lines and then miR-140-5p expression levels were tested. We found that the expression of miR-140-5p was significantly inhibited in anti-miR-140-5p group (Fig. 2G). Next, reporter constructs containing either the wild-type (WT) HMGN5 3′-UTR or HMGN5 3′-UTR with mutation (MT) at the predicted miR-140-5p target sequence were cotransfected into osteosarcoma U2-OS cells and then transduction of Mimic NC, miR-140-5p mimics, anti-NC or anti-miR-140-5p. Luciferase reporter assays showed that miR-140-5p mimics significantly decreased the luciferase activity of the WT HMGN5 3′-UTR by approximately 37.6% in U2-OS cells relative to the control, whereas miR-140-5p inhibition by anti-miR-140-5p substantially increased luciferase activities of WT HMGN5 3′-UTR compared with anti-NC (p < 0.05, Fig. 2H). However, it had no effect on MT HMGN5 3′-UTR (Fig. 2I). Taken together, these results demonstrated that HMGN5 is the direct target of miR-140-5p.

### Overexpression of HMGN5 increased chemoresistance and activated autophagy in U2-OS/miR-140-5p cells

**Figure 3:**
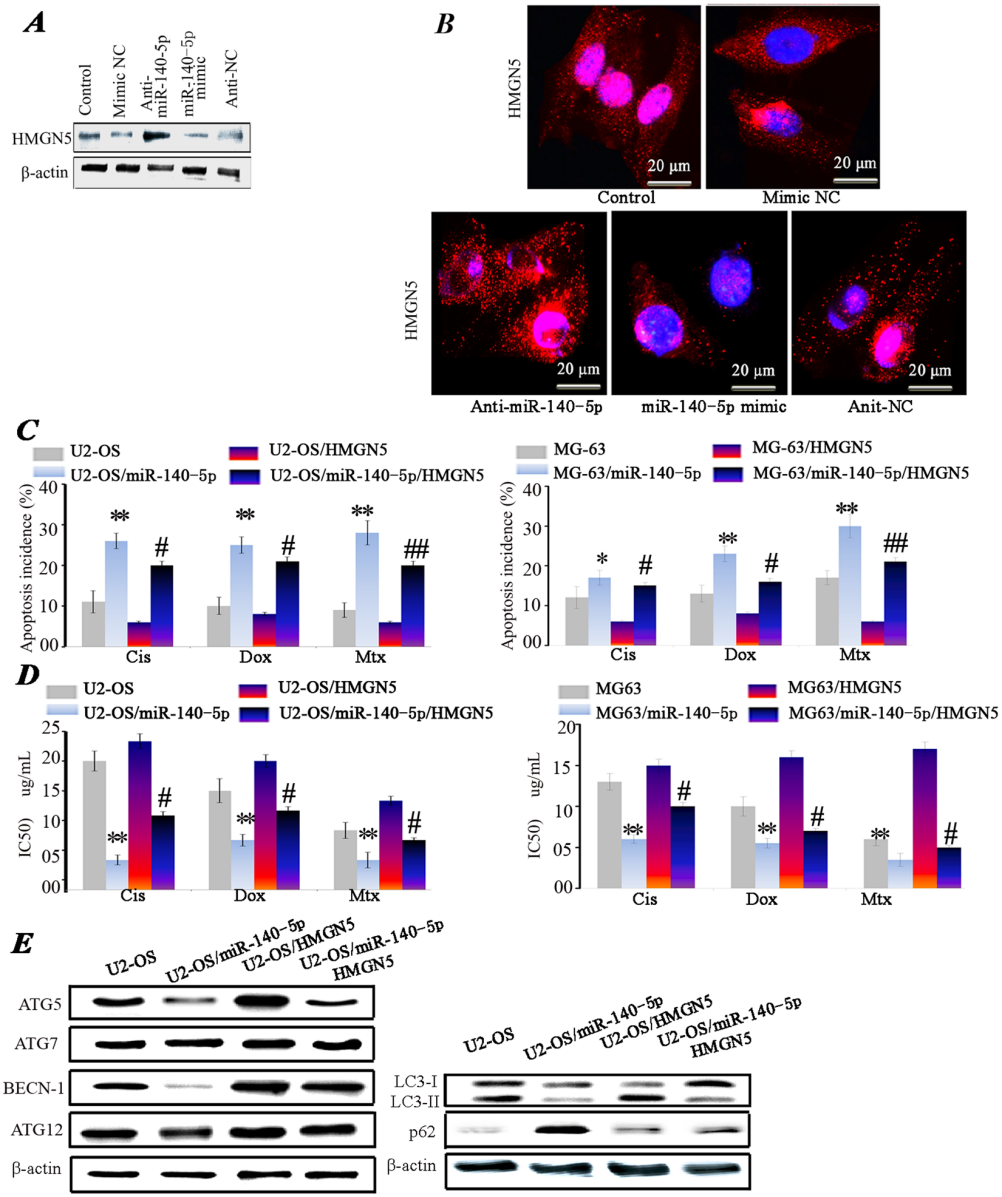
Overexpression of HMGN5 increased chemoresistance and activated autophagy. HMGN5 expression level was evaluated using Western blot (**A**) and Confocal laser scanning microscopy (CLSM) (**B**). Blue fluorescence indicates nuclei stained with DAPI, and red fluorescence indicates the HMGN5 protein. *P < 0.05, **P < 0.01 vs control. The apoptosis incidence (**C**) and IC50 values (**D**) in U2-OS and MG-63 cells transfected with miR-140-5p in combination with or without Lenti-HMGN5 vector. (**E**) Expression levels of ATG5, ATG7, ATG12, BECN-1, p62 and LC3-II/I proteins in cells transfected with miR-140-5p in combination with or without Lenti-HMGN5 vector. (*P < 0.05, **P < 0.01 U2-OS vs U2-OS/miR-140-5p; ^#^P < 0.05, ^##^P < 0.01 U2-OS/miR-140-5p vs U2-OS/miR-140-5p/HMGN5). The results of p62, LC3-II/I and β-actin were gotten from the second experiment.

To further confirm that miR-140-5p modulates the expression of HMGN5, U2-OS cells were transfected with mimic NC, miR-140-5p mimics, anti-NC or anti-miR-140-5p. Subsequently, HMGN5 protein expression was analyzed by Western blot (Fig. 3A) and confocal laser scanning microscopy (Fig. 3B). Transfection of the miR-140-5p mimics significantly decreased the protein expression level of HMGN5, whereas anti-miR-140-5p had the opposite effects (Fig. 3A). Consistently, confocal laser scanning microscopy indicated that HMGN5 were down-regulated in U2-OS/miR-140-5p cells compared with U2-OS cells (Fig. 3B).

Next, to investigate whether miR-140-5p regulated the chemoresistance of osteosarcoma cells, we established U2-OS/miR-140-5p cells and MG63/miR-140-5p that stably expressed miR-140-5p by lentivirus system. Remarkably, overexpression of miR-140-5p increased the apoptosis induced by chemotherapy agents (Fig. 3C). Given that HMGN5 was the target of miR-140-5p, we applied lentivirus system to over expression HMGN5 to explore the relationship between HMGN5 and miR-140-5p in the chemoresistance of osteosarcoma. As expected, miR-140-5p sensitized the U2-OS and MG63 cells to chemotherapeutic agents while overexpression of HMGN5 could reverse the effect of miR-140-5p (Fig. 3C). Consistently, miR-140-5p was able to cause a decrease of IC50 values for these three chemotherapeutic agents. However, HMGN5 could reverse the effect of miR-140-5p (Fig. 3D).

HMGN5 is a member of the HMG box family and acts as damage associated molecular pattern molecule. HMGN5 is also involved in autophagy in osteosarcoma as shown by our previous report^19^. To further explore the function of miR-140-5p, the markers of autophagy were also assessed in U2-OS/miR-140-5p cells. BECN-1, ATG5 and LC3-II/I proteins expression levels were significantly decreased in U2-OS/miR-140-5p cells compared with U2-OS cells, Whereas p62 were significantly increased in U2-OS/miR-140-5p cells. Interestingly, we found that the overexpression of HMGN5 led to the increased expression level of BECN-1, ATG5, LC3-II/I proteins and a decrease of p62 protein, whereas the expression of Atg12 and ATG7 was not markedly altered by HMGN5 overexpression (Fig. 3E). These data suggest that p62, BECN-1, ATG5 and LC3 are the downstream targets of HMGN5. Thus they are direct functional target genes of miR-140-5p.

### miR-140-5p suppressed autophagy in osteosarcoma cells

**Figure 4:**
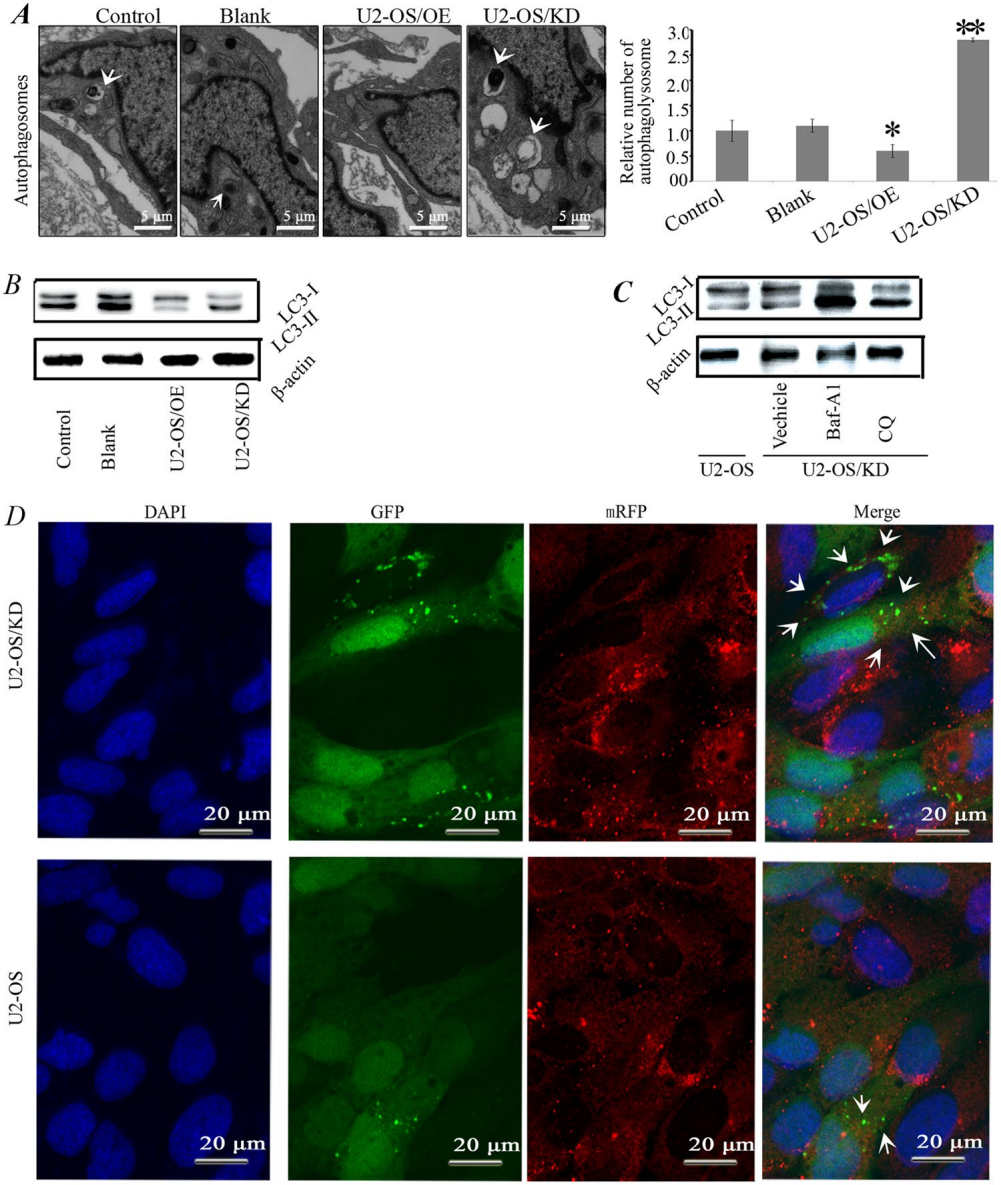
miR-140-5p suppressed autophagy in osteosarcoma cells. (**A**) Autophagy was evaluated in a blank group (untransfected cells), a control group (cells transfected with the control lentivirus), an OE group (overexpression of miR-140-5p) and a KD group (knocking down of miR-140-5p). Using TEM. The white arrows indicated autophagosomes. (**B**) Western blot analysis of the expression of LC3-II and LC3-I for U2-OS cells in control, blank, OE and KD groups. (**C**) Western blot analysis of the expression of LC3-II and LC3-I. Knocking down of miR-140-5p U2-OS cells (U2-OS/KD) were incubated with 20 µM Choroquine or 10 nM Baf A1 for 12 hrs and then assessed by Western blot. (**D**) U2-OS/KD cells stably expressing *mRFP-GFP-LC3* and then examined using confocal microscopy. The white arrows indicate autophagosomes.

To further confirm the role of miR-140-5p in regulating autophagy in osteosarcoma cells, we applied lentivirus system to make stable cell lines including a blank group (untransfected cells), a control group (cells transfected with the control lentivirus), an OE group (overexpression of miR-140-5p) and a KD group (knocking down of miR-140-5p). Autophagosomes were evaluated using transmission electron microscopy (TEM), confocal microscopy and Western blot. TEM revealed a marked accumulation of autophagosomes in the cytoplasm of U2-OS/KD cells compared with U2-OS cells (Fig. 4A). Autophagic vacuoles are denoted by arrows.

Next, we investigated the effect of miR-140-5p on the expression of LC3-I and LC3-II. Results showed that miR-140-5p overexpression significantly reduced the conversion of LC3-I to LC3-II, while knocking down of miR-140-5p induced autophagy, with an increase in the conversion of LC3-I to LC3-II (Fig. 4B). To further confirm the increased autophagic flux, we examined changes in autophagic flux by comparing the levels of LC3-II in the presence and absence of the lysosome inhibitor chloroquine (CQ) and Bafilomycin A1 (Baf A1). Increased LC3-II expression and an accompanying increase in the conversion of LC3-I to LC3-II were clearly detected in U2-OS/KD compared with U2-OS cells (Fig. 4C). More importantly, CQ or Baf-A1 treatment significantly increased endogenous LC3-II accumulation (Fig. 4C). Therefore, the conversion of LC3-I to LC3-II was up-regulated after CQ or Baf-A1 treatment, confirming increased autophagic flux in U2-OS/KD cells. To further validate these results, we established a U2-OS/KD cell model that stably expresses an *mRFP-GFP-LC3* fusion protein. U2-OS/KD cells showed a higher *mRFP-GFP-LC3* signal than parental U2-OS cells, indicating that autophagy is enhanced when osteosarcoma cells knocking down of miR-140-5p (Fig. 4D).

### Inhibition of autophagy restored the chemosensitivity of U2-OS/KD

**Figure 5:**
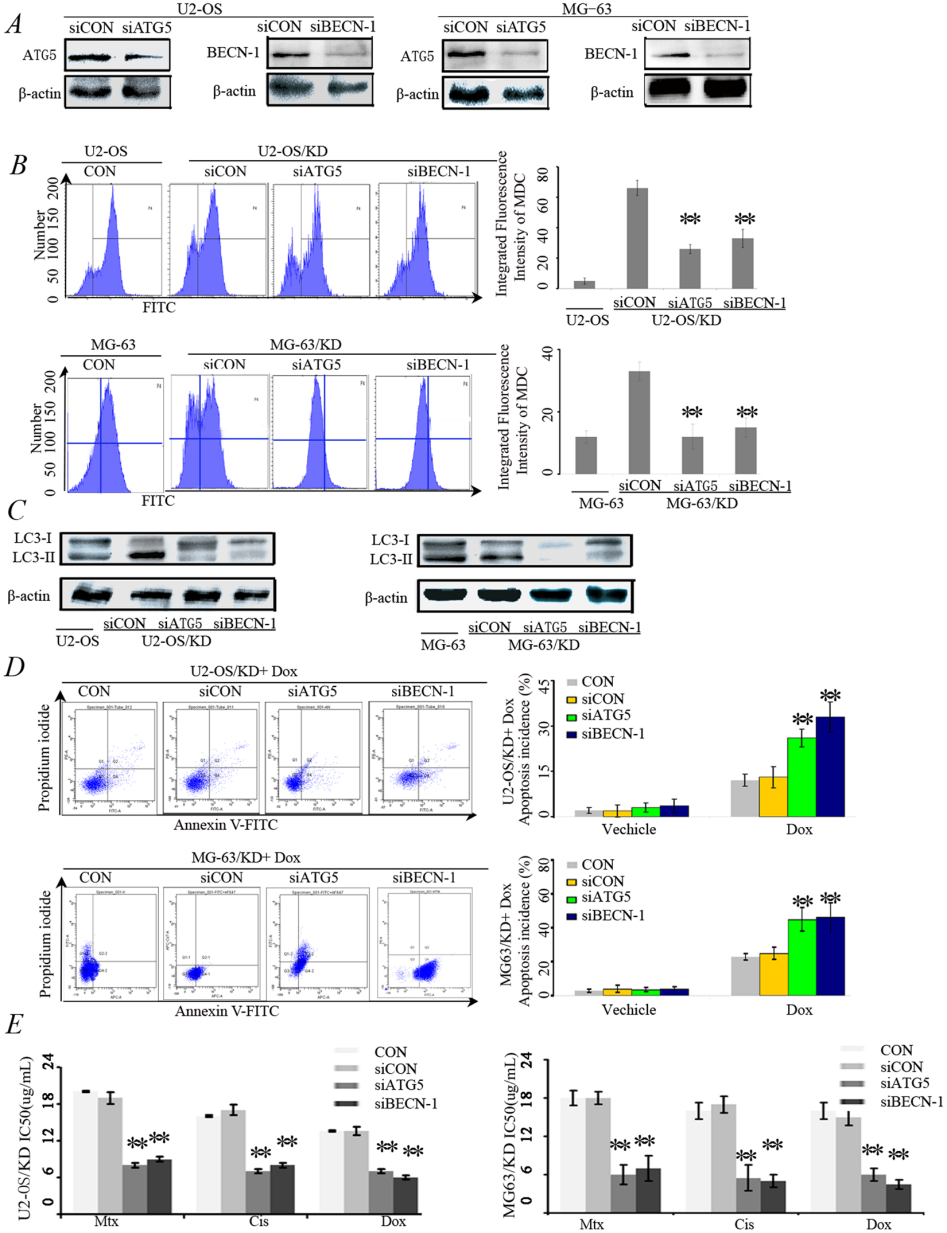
Inhibition of autophagy restored the chemosensitivity of U2-OS/KD and MG-63/KD cells. (**A**) Western blot analysis for the expression of BECN-1 and ATG5 proteins. U2-OS/KD cells and MG-63/KD were co-transfected with BECN-1 siRNA (siBECN-1) or ATG5 siRNA (siATG5). After 48 hrs, BECN-1 and ATG5 proteins were detected using Western blot. (**B**) Flow cytometry assay to detect autophagy level using by MDC staining. Cells were described as (**A**). (**C**) Western blot analysis for the expression of LC3-II/I proteins. (**D**) Flow cytometry for apoptosis analysis using Annexin V-FITC/PI double staining. U2-OS/KD cells and MG-63/KD were co-transfected with siBECN-1 or siATG5. After 48 hrs, the cells were treated with 1 μM Dox for 24 hrs. **P < 0.05 versus U2-OS/KD cells treated with Dox.

We have confirmed that overexpression of HMGN5 decreased the sensitivity of U2-OS/miR-140-5p and MG63/miR-140-5p cells to anticancer agents (Fig. 3C,D), as well as increased autophagy (Fig. 3E). The next question was to investigate whether autophagy truly contributed to miR-140-5p down-regulation mediated chemoresistance in osteosarcoma cells. Autophagy was inhibited by knocking-down of ATG5 (Fig. 5A, left panel) or BECN-1 (Fig. 5A, right panel), and then the effects of chemotherapy were assessed.

As shown in Fig. 5B, MDC staining indicated that autophagy was markedly decreased in both U2-OS/KD and MG63/KD cells that transfected with siRNAs targeting BECN-1 or ATG5 (Fig. 5B). Consistently, decreased conversion of LC3-I to LC3-II proteins were also observed in siATG5 or siBECN-1 cells (Fig. 5C).

Fig. 5D showed that either knocking down BECN-1 or ATG5 enhanced the sensitivity of U2-OS/KD and MG63/KD cells to doxorubicin (Dox) (Fig. 5D). Consistently, knocking down of BECN-1 or ATG5 decreased the IC50 values for the three chemotherapeutic agents (Fig. 5E). These data suggested that U2-OS/KD and MG63/KD cells exhibit chemoresistance by up-regulating autophagy.

### miR-140-5p was associated with chemoresistance and increased the chemoresistance

**Figure 6:**
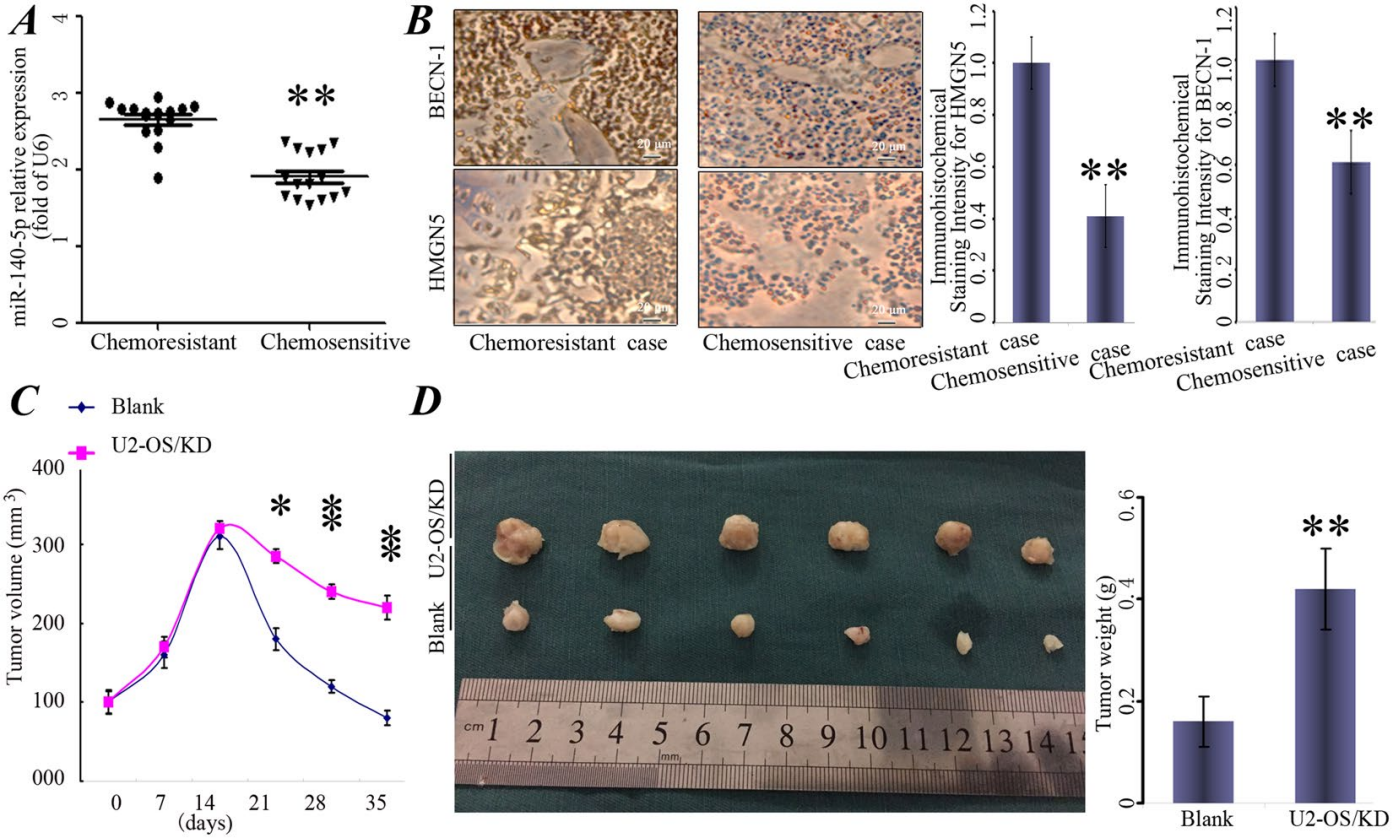
miR-140-5p is associated with chemoresistance and increased the chemoresistance. (**A**) miR-140-5p expression was assessed in chemosensitive patients (n = 15) and chemoresistant patients (n = 15). (**B**) Representative photographs of IHC stained human osteosarcoma tissue specimens at a magnifcation of 200x. HMGN5 and BECN-1 were markedly up-regulated in chemoresistant patients. Columns, mean of intensity of the staining (n = 15). (**C**) The tumor volumes were calculated as V = length × width^2^/2 and measured at 1, 2, 3, 4, 5 weeks time points. (**D**) Representative photos for tumor isolated from the nude mice (n = 6). U2-OS/KD cells or control cells (Blank) were transplanted in the left and right side of the mice, respectively. P < 0.05, **P < 0.01 vs Blank.

To further verify the function of miR-140-5p in clinical samples, qRT-PCR and immunohistochemistry were used to detect miR-140-5p and the expression of HMGN5 and BECN-1 expression was detected in tissues from 15 cases of patients with relapsed osteosarcoma by immunohistochemistry (chemoresistant) and 15 cases of chemosensitive patients. We found that the Expression levels of miR-140-5p were decreased in 15 patients with chemoresistant, and the expression of HMGN5 and BECN-1 were significantly up-regulated in these chemoresistant patients compared with chemosensitive patients (Fig. 6A,B).

Moreover, to further determine the effects of miR-140-5p on tumor chemoresistance, nude mice were subcutaneously injected with U2-OS/KD cells or control cells (Blank) and the animals were monitored closely for tumor growth. As shown in Fig. 6C, there was no significant difference in tumor size between miR-140-5p-knocking-down cells (U2-OS/KD) and those in cells expressing the control vector within 2 weeks. Importantly, after expose to Dox for 3 weeks, miR-140-5p-knocking-down tumors were bigger in size and heavier in weight compared to the controls (Fig. 6D).

## Discussion

In this study, by using miRNA microarrays analysis, we identified 23 differentially expressed miRNAs in human osteosarcoma samples compared with normal bone samples. We found that miR-140-5p frequently highly expressed in human osteosarcoma samples. Moreover, the correlation between the expression of miR-140-5p and the tumor size, clinical stage, recurrenceand distant metastasis of osteosarcoma was also analyzed. To further investigate the role of miR-140-5p, gain- and loss-of-function approaches revealed that miR-140-5p in osteosarcoma enhanced the chemoresistance by regulating autophagy. The function of miR-140-5p was further confirmed in mouse tumor models. In addition, we discovered a novel mechanism of autophagy regulated by non-coding miR-140-5p in osteosarcoma through direct binding at the 3′-UTR of the HMGN5 mRNA. HMGN5, which widely expressed in eukaryotes, regulates the growth, and differentiation of various types of cells^31^ and our previous results demonstrated that HMGN5-mediated autophagy contributed to chemoresistance in osteosarcoma cells. Our results extended previous observations and found that miR-140-5p in osteosarcoma enhanced the chemoresistance by up-regulating HMGN5-mediated autophagy, suggesting that miR-140-5p is a potential target of an adjuvant therapy for osteosarcoma patients.

Previous studies demonstrated that miR-140-5p functions as both a tumor suppressor and a tumor promoter in cancers. Recent research found that miR-140-5p serves as a tumor suppressor by inhibiting ovarian cancer via repression of PDGFRA^25^. However, our studies found that miR-140-5p facilitated the chemoresistance of osteosarcoma through regulating HMGN5-mediated autophagy, promoting tumor in osteosarcoma. This result suggests that the function of miR-140-5p may be disease specific. Consistent with our findings, miR-140-5p has also been reported to participate in the regulatory network for cancer invasion in other cancer types^28, 32^. We showed that HMGN5 is the direct target of miR-140-5p and overexpression of miR-140-5p in osteosarcoma cell lines increased the osteosarcoma cell chemoresistance. Our studies further revealed that the increase of miR-140-5p–mediated chemoresistance was directly due to the up-regulation of HMGN5. By utilizing siRNA against HMGN5, we found that i depletion of HMGN5 could reverse the effect of miR-140-5p and lead to increase of chemosensitivity in osteosarcoma cell lines. Consistently, miR-140-5p has been reported to participate in the regulatory network of autophagy in colorectal cancer cells^27^.

The clinical relevance of miR-140-5p was clearly demonstrated by quantification of the miR-140-5p’ expression levels in a set of 40 osteosarcoma patient samples of primary tumor tissues and ANCT. As expected, we found that miR-140-5p was progressively up-regulated in osteosarcoma tumor tissues and further increased in the chemoresistance osteosarcoma patients, suggesting that miR-140-5p is significantly correlated with osteosarcoma chemoresistance. In addition to HMGN5 and chemoresistance, our work revealed a novel regulatory function of miR-140-5p in osteosarcoma and autophagy.

Autophagy, a mechanism that delivers aging organelles and damaged components to lysosomes for degradation, could facilitate cell survival by supplying nutrients to stressed cancer cells^33^, and promote cancer invasion through the TGF-β/Smad2 signaling pathway^27, 34, 35^. Furthermore, mounting evidences had suggested that activation of autophagy promoted chemoresistance^8, 36, 37^. On the other hand, we found that siRNA knocking down of BECN-1 or ATG5 could reduce the chemoresistance and reversed the effect of miR-140-5p, suggesting an important role of autophagy on chemoresistance and miR-140-5p increased the chemoresistance through miR-140-5p/HMGN5/autophagy regulatory loop.

Many studies have been carried out to investigate the potential role of miRNAs in osteosarcoma, which provides new anti-osteosarcoma therapeutic regimens. For example, researchers found that low expressions of miR-133b/miR-503 were correlated with shorter overall survival of osteosarcoma patients^38^. miR-20a promotes the proliferation of human osteosarcoma cells by suppressing growth response 2 expression^39^. More importantly, it is reported that transfection of miR-202 mimics into osteosarcoma cells significantly promotes chemoresistance by targeting PDCD4 while transfection of miR-202 inhibitor enhances the chemosensitivity. In addition, it had been shown that several miRNAs regulated cancer progression through autophagy, such as miR-502^40^, miR-30a^41^, miR-34a^42^, miR-4487 and miR-595^43^.

Our research is the first evidence demonstrating that miR-140-5p has an effect on chemoresistance of osteosarcoma cells. The Kaplan–Meier analysis in our study revealed that the prognosis of osteosarcoma patients was significantly related to the miR-140-5p expression level. However, these results just provide very limited evidence due to a high variability in miRNA expression levels, inter-individual and race variation. Furthermore, the use of osteosarcoma cells from two cell line still very limited. Similarly, the lentiviral based delivery approach is also not ideal for therapeutic development., To confirm the role of miR-140-5p, we should use more cell lines and primary tumor in the future. On the other hand, we need to develop a more effective and optimized method to deliver miR-140-5p inhibitor *in vivo*. In addition, much more work need to be performed to elucidate the reason that promoted down-regulation of miR-140-5p. Our further study aimed to clarify the underlying mechanism of dysregulation of miR-140-5p in osteosarcoma chemoresistance. Further study, which using more cell lines and primary tumor is therefore indispensable to confirm the findings of this study.

In conclusion, we found that miR-140-5p promoted chemoresistance through the up-regulation of HMGN5, which is a key player in transcriptional activation by interacting with nucleosomes^44^. miR-140-5p promoted chemoresistance by regulating HMGN5-mediated autophagy *in vitro and vivo*. We identified a novel mechanism of miR-140-5p as a critical regulator of autophagy in osteosarcoma. As many miRNA based therapeutics are currently in clinical trials, our results suggest that hsa-miR-140-5p could be a valuable chemotherapy strategy for treating osteosarcoma patients.

## Materials and Methods

### Ethics approval

All research involved human tissue samples was approved by the Ethics Review Committee of Second Military Medical University Shanghai and written informed consent was obtained from all participating patients. All participants provided written informed consent for participation in this study and the methods were carried out in accordance with the approved guidelines. All animal experiments were carried out in “accordance” with the relevant guidelines.

### Tissue specimens

Forty osteosarcoma patients who received neoadjuvant chemotherapy and underwent surgery were included in Shanghai Changzheng Hospital between January 2011 and January 2014. Eligibility criteria for entry onto the study were diagnosis of primary, central, high-grade osteosarcoma of the extremity who received neoadjuvant chemotherapy and underwent surgery; and normal hepatic, renal, bone marrow, and cardiac function. The TNM and histological classification were performed according to World Health Organization criteria. After screening, the chemoresistance cohort consists of eight osteosarcoma patients who showed <90% tumor necrosis (mean 58.2% ±3.2%) after neoadjuvant chemotherapy and were defined as poor responders. Another tumor stage-matched osteosarcoma patients, who showed ≥90% tumor necrosis (mean 96.1% ± 4.1%) as good responders^45^, were enrolled as chemosensitive controls (n = 7). All patients were treated preoperatively with neoadjuvant chemotherapy, the cumulative doses of medicines as follows: doxorubicin 420 mg/m^2^, methotrexate 120 g/m^2^, cisplatin 600 mg/m^2^. All tumor specimens and the adjacent non-cancer tissues (ANCT) were immediately frozen in liquid nitrogen after resection and stored at −80 °C. All 40 osteosarcoma patients received follow-up and the median follow-up was 32 months (range, 6–38 months).

### miRNA Microarrays analysis

miRNA microarrays analysis was outsourced to RiBio Cor (Ribobio, Guangzhou, China). Briefly, total RNA extract was purified using mirVana™ miRNA Isolation Kit (Ambion, USA) from the tumor specimen before and after chemotherapy and then labeled and hybridized using the Agilent miRNA Complete Labeling and Hybridization Kit (Agilent Technologies, San Diego, USA). The labeled RNA was performed with the Axon GenePix 4000B microarray scanner. miRNA signal intensities were log2 transformed, and analyzed for differentially expressed miRNAs by using the significance analysis of microarrays (SAM, version 3.01). We selected miRNAs whose expression levels changed at least two fold between the tumor specimen before and after chemotherapy for further analysis.

### Cell culture and transfection

Human hFOB1. 19 osteoblast and Human osteosarcoma MG-63 and U-2 OS cell lines were reserved in our laboratory as described previously^46^. Human MG-63, and U-2 OS cell lines were cultured in high glucose DMEM medium supplemented with 10% fetal bovine serum (FBS, HyClone, USA), 100 U/mL penicillin, and 100 μg/mL streptomycin and maintained at 37 °C and 5% CO_2_. MG-63 and U2-OS cells were transiently transfected with 100 nmol/L of miR-140-5p mimics, mimics NC, miR-140-5p inhibitor, anti-miR-140-5p, or miRNA negative control (anti-NC), (Ribobio, Guangzhou, China) using Lipofectamine 2000 (Life Technology, CA, USA). All of the RNA oligoribonucleotides were purchased from Ribobio (Ribobio, Guangzhou, China).

For stable transfection, U2-OS or MG-63 cells suspension were plated at 2 × 10^5^ cells/well in 6-well attachment plates. 24 hrs after plating, U2-OS cells were transfected with lentiviral vectors expressing miR-140-5p (U2-OS/miR-140-5p) or a blank lentivirus expression vectors (CON) (GeneChem, Shanghai, China). After incubating for 72 hrs, the efficiency of transfection was examined by miR-140-5p qRT-PCR. siRNAs against HMGN5, ATG5 or BECN-1 (Santa Cruz, CA, USA) were transfected to the U2-OS/miR-140-5p following the manufacturer's protocol. Scrambled siRNA ((Life Technology, CA, USA) was transfected as negative controls (siCON). The transfection efficiency was quantified by Western blot.

### The 50% cytotoxic concentration (IC50) values in osteosarcoma cells

The four osteosarcoma cell lines (mimic NC, miR-140-5p mimics, anti-NC or anti-miR-140-5p) were seeded into wells of a 96-well plate at a density of 1 × 10^4^ cells/well in medium containing 10% FBS. After 24 hrs incubation, cells were washed twice with the medium and exposed to a range of chemotherapeutic agents for 48 hrs. Subsequently, cells were detached by trypsinization and the viable cell population was determined using 3-(4,5-dimethylthiazol-2-yl)-2,5-diphenyltetrazolium bromide. The 50% cytotoxic concentration (IC50) values were defined as the chemotherapeutic agents concentration producing 50% inhibition of cell growth. Similarly, the IC50 values of transfected cell lines (miR-140-5p, HMGN5 and miR-140-5p/HMGN) and controls (untransfected) were also determined as described above.

### Quantitative real-time PCR (qRT-PCR)

Total RNA was extracted from cells or surgical specimens using TRIzol reagent (Life Technology, CA, USA). To detect the expression of miR-140-5p, qRT-PCR was performed using a Reverse Transcription Kit Ribobio (Ribobio, Guangzhou, China) according to the manufacturer's instructions. Specific qRT-PCR primers for miR-140-5p and U6 snRNA were purchased from Ribobio (Ribobio, Guangzhou, China). Each sample was analyzed in triplicate. U6 snRNA was used as the endogenous control. The fold-change in gene expression was calculated by 2^−ΔΔCT^ method.

### MicroRNA Target Prediction

To predict the target genes of miR-140-5p, bioinformatics algorithms TargetScan (http://www.targetscan.org), miRBase (http://www.mirbase.org/) and TargetScan (http://www.Targetscan.org/) was used.

### Luciferase reporter assay

Luciferase reporter assay was performed as described previously^47^. The PCR fragment of the 3′UTR of HMGN5 containing predicted binding sites of miR-140-5p was cloned from human genomic DNA and inserted into pGL3-control vector (Promega Corporation, WI, USA). 24 hrs before transfection, 1.5 × 10^4^ U2-OS cells were plated in a 96-well plate. 10 pmole of miR-140-5p mimics, mimic NC, miR-140-5p inhibitor, Anti-miR-140-5p or Anti-NC was transfected into cells together with 100 ng of pGL3-3UTR HMGN5 and 1 ng of Renilla luciferase plasmid pRL-SV40 (Promega Corporation, WI, USA). Luciferase assays were performed 24 hrs after transfection by the dual-luciferase reporter assay system.

### Western blots

Cells were collected and lysed in 1 × RIPA buffer (Sigma, MO, USA) and then subjected to Western blot as described previously^46^. 80 µg of each protein extract was separated by 6~15% SDS-PAGE gels and transferred to nitrocellulose membrane. The immunoreactive bands were firstly incubated with the primary antibodies, including HMGN5 (1:500), ATG5 (1:1000), BECN-1 (1:2000), and β-actin (1:2000) (Cell Signaling Technology, MA, USA). Then, the immunoreactive bands were incubated with the secondary antibody and visualized using ECL-PLUS Kit (Beyotime Institution of Biotechnology, Haimen, China).

### Electron microscopy

Cells were fixed in 2.5% glutaraldehyde for 2 hrs at room temperature, then incubated in 1% osmium tetroxide for 1 hr. After dehydration in an increasing concentration gradient of ethanol, the samples were embedded in Durcopan ACM for 6 hrs, then samples were cutted into 50-nm sections and stained with 3% uranyl acetate and lead citrate. Images were generated using a transmission electron microscope (Philips CM, Amsterdam, Netherlands).

### Stably expressing of mRFP-GFP-LC3

Adenovirus vector containing the *mRFP-GFP-LC3* reporter was purchased from Hanbiology (Hanbio, Shanghai, China). Autophagy level was examined as previously described using confocal microscopy (Olympus, Tokyo, Japan)^19^. Briefly, up to 24 hrs recovery after transfection, the cells samples were treated according to the protocol and then analyzed using confocal microscopy.

### Immunofluorescence staining

Immunofluorescence staining was performed following standard protocol. Briefly, U2-OS cells were fixed with 4% paraformaldehyde, permeabilized with 1% Triton X-100 and then blocked with 10% goat serum. Subsequently, the cells were then incubated with primary antibodies recognizing HMGN5 (1:100; Abcam, Cambridge, UK) overnight, and then incubated with AffiniPure donkey anti-rabbit IgG (1:200, red fluorescence) for 1 hr at room temperature. 4′,6-diamidino-2-phenylindole (DAPI, Beyotime Institution of Biotechnology, Haimen, China) was applied to stain the nuclei for 5 mins. The gene expressions were analyzed using confocal microscopy.

### Monodansycadaverine (MDC) staining

MDC (Sigma, MO, USA) is a marker for autolysosomes and the autophagy levels were qualitatively evaluated by flow cytometry using monodansycadaverine staining^48^. U2-OS transfected with or without miR-140-5p were incubated with 0.05 mM MDC staining for 10 mins at 37 °C and determined as the percent of MDC positive cells automatically analyzed using FlowJo software (Tree Star, CA, USA).

### Apoptosis assays

Cells in each group were treated with Dox (1 µM), Cis (20 mmol/L), or Mtx (50 mmol/L) for 12 hrs and the apoptosis level was analyzed by Flow cytometry. Flow cytometry was performed as described previously^46^. Harvested cells were resuspended in 500 µL binding buffer and then stained with 5 µL FITC-Annexin-V (BD Biosciences, CA, USA) and 5 µL propidium iodide (BD Biosciences, CA, USA) for 30 mins in the dark at 4 °C. Cells were analyzed by flow cytometry within 1 hr.

After 24 hrs incubation, the transfected cell lines (miR-140-5p, HMGN5 and miR-140-5p/HMGN) and controls were treated with Dox (1 µM), Cis (20 mmol/L), or Mtx (50 mmol/L) for 12 hrs and the apoptosis were detected as described above.

### Immunohistochemical analysis (IHC)

Tissue specimens were immediately obtained from patients with relapsed osteosarcom at Shanghai Changzheng Hospital and sent to a pathology department for diagnosis by three pathologists based on histopathological evaluation. As a result, 15 cases of chemoresistance and 15 cases of chemosensitive patients were enrolled in this program. For IHC analyses, paraffn-embedded samples were deparaffnized with xylene and rehydrated through addition of graded ethanol. The samples were incubated with the antibody at a dilution of 1:100 overnight, followed by incubation with biotinylated secondary antibody (1:500 dilutions). Finally, the immunohistochemistry results were scored by both the percentage of positive detection and intensity of the staining.

### *In vivo* Chemosensitivity assay

6-week-old female nude mice were maintained under specifc pathogen-free conditions at Second Military Medical University Shanghai. Animal experiment was performed to evaluate the tumor growth and the sensitivity of osteosarcoma cells to chemotherapy *in vivo*. In brief, 1.0 × 10^7^ U2-OS cells stably transfected with Lenti-miR-140-5p (U2-OS/KD) or lenti-NC (Blank) were harvested by treatment with trypsin-EDTA (Invitrogen) and resuspended in PBS. Then osteosarcoma cells suspension (U2-OS/KD group) were subcutaneously transplanted in the left proximal tibia of each anesthetized nude mice (n = 6 animals/group) and the Blank cells group were injected into in the right proximal tibia (n = 6). Two weeks later, the mice were intraperitoneally injected with PBS containing doxorubicin (5 mg/kg) twice per week according to a previous study^49^. After inoculation, tumor size was measured every 7 days, and tumor volumes (mm^3^) were calculated using the formula: V = length × width^2^/2. The mice were sacrificed on day 35, and the tumors were measured and photographed 35 days after inoculation. All animal experiments were approved by the Animal Care and Use Committee of Second Military Medical University Shanghai.

### Statistical analysis

One-way analysis of variance (ANOVA) was used to analyze the differences between groups. The LSD method of multiple comparisons was used when the probability for ANOVA was statistically significant. P-values < 0.05 was considered statistical significant. Survival curve was estimated using the Kaplan–Meier method, and the differences in survival distributions were evaluated by the Log–rank test. All statistical analyses were performed using the SPSS version 17.0 software package (SPSS Inc., IL, USA).
